# Correlation between anti-Müllerian hormone, age, and number of oocytes: A retrospective study in a Brazilian *in vitro* fertilization center

**DOI:** 10.5935/1518-0557.20210083

**Published:** 2022

**Authors:** Isadora Ferreira Kozlowski, Matheus Campos Carneiro, Vinicius Bonato da Rosa, Alessandro Schuffner

**Affiliations:** 1 Faculdade de Medicina, Pontifícia Universidade Católica Curitiba, Paraná, Brazil; 2 Faculdade de Medicina, Faculdade Evangélica Mackenzie, Curitiba, Paraná, Brazil; 3 Conceber Centro de Medicina Reprodutiva, Curitiba, Paraná, Brazil

**Keywords:** AMH, aging, assisted reproductive technology

## Abstract

**Objective:**

Is the AMH level correlated with age and number of mature oocytes retrieved from stimulated cycles?

**Methods:**

This descriptive, retrospective, observational study included the data of about 1500 patients submitted to Assisted Reproductive Technology treatments in a clinic in Brazil between July 2012 and April 2019. Patients not submitted to IVF and/or without AMH level records were excluded. The study included women with fertility issues aged 20-50 years submitted to IVF. A total of 733 patients were included. The patients were divided by age into three groups (≤35 years old; 36-39 years old; ≥40 years old).

**Results:**

The mean AMH concentration ranged from 2.65 to 1.35 ng/mL and was significantly different between the groups. The mean total number of retrieved oocytes ranged from 9.5 to 5.42 and was significantly different between the groups. The mean number of mature oocytes ranged from 7.14 to 4.58. There was no significant difference in the number of mature oocytes between patients aged 36-39 years and ≥40 years. Negative correlations were observed between patient age and total number of retrieved oocytes (-0.3354) and number of mature oocytes (-0.2839). AMH was negatively correlated with age (-0.3257), although positive correlations with total number of oocytes (0.6702) and number of mature oocytes (0.5770) were observed.

**Conclusions:**

This is the largest study performed with Brazilian patients to correlate AMH levels, age, number of oocytes, and number of mature oocytes from controlled ovarian stimulation cycles. Our data showed that as age increases, AMH levels, number of retrieved oocytes, and number of mature oocytes decrease significantly. However, no significant difference in number of mature oocytes was observed when patients aged 36-39 and ≥40 years were compared. In addition, a positive correlation was found between serum AMH levels and total number of retrieved and mature oocytes from stimulated cycles.

## INTRODUCTION

Infertility is of great medical importance and produces relevant socioeconomic impact, as it affects more than 186 million people worldwide. Factors directly related to this condition range from female age and previous illnesses to the unwanted period of inability to conceive (Jacobson *et al*., 2018).

One of the most used methods in high complexity assisted reproduction is intracytoplasmic sperm injection (ICSI), which has become a viable option with high chances of success for patients seeking conception. The level of anti-Müllerian hormone (AMH) has become an important element in the initial investigation of female patients and is of great importance throughout the process (Jacobson *et al*., 2018). AMH is a glycoprotein produced by granulosa cells in primary, preantral and small antral follicles, and its clinical applicability has been demonstrated in several studies (Ebner *et al*., 2006; La Marca & Volpe, 2006; Gomez *et al*., 2016). In the literature, there is strong evidence that AMH is a suitable indicator to assess ovarian reserve and predict the probability of achieving pregnancy (Nelson *et al*., 2009; Peluso *et al.*, 2014; da Silva *et al*., 2014; Gomez *et al*., 2016). Ovarian reserve is defined as the number of ovarian follicles that might be available for use in fertilization, representing a woman's fertile ovarian potential (Nelson *et al*., 2009; Romão & Navarro, 2013; Peluso *et al*., 2014; da Silva *et al*., 2014; Gomez *et al*., 2016). Thus, the assessment of ovarian reserve assumes an important role in the attempt to estimate reproductive capacity, which needs to be calculated and informed to patients before treatment initiation.

An association has been established between poor ovarian response and decreasing levels of AMH. AMH levels decrease substantially with age, which consequently leads to considerably lower probabilities of achieving of pregnancy (La Marca & Volpe, 2006; Visser *et al*., 2006; Tal & Seifer, 2017). According to a study published by Khan *et al*. (2019), AMH levels decrease by about 6% a year in older women, showing the direct relationship existing between increased female age and declines in follicular supply. However, female age is not an absolute predictor of reproductive capacity (Khan *et al*., 2019). In this context, AMH appears as an excellent predictor of fertility.

A study developed by the POSEIDON (Patient Oriented Strategies Encompassing Individualize Oocyte Number) group proposed a new stratification in assisted reproduction. In the study, the ideal AMH level used for comparison was 1.2 ng/mL, based on the idea that women with at least this level of AMH might have better outcomes in terms of number of oocytes generated. This assumption was confirmed in the study, which showed an association between AMH and female age (Humaidan *et al*., 2016).

Although AMH is primarily related to the quantity rather than the quality of oocytes, the higher the AMH levels, the greater are the expectations around the availability of oocytes and ultimately embryos for transfer (Anderson *et al*., 2012).

Patients must be informed at the beginning of treatment about their ovarian reserve and the potential associations with age, so that they develop more realistic expectations about their response to ovulation induction treatment and become aware of the strong relationship between AMH levels and the probability of achieving pregnancy (Nelson *et al*., 2009; Peluso *et al*., 2014; da Silva *et al*., 2014; Gomez *et al*., 2016). Research in this area is of great importance and consequence, since it may lead to better approaches based on each patient's profile. This study aimed to find whether there is an association between AMH levels, age, and number of retrieved and mature oocytes of Brazilian patients undergoing assisted reproductive technology (ART) treatments.

## MATERIALS AND METHODS

This descriptive retrospective observational study included about 1,500 electronic medical records of patients treated at an ART clinic in Curitiba, Paraná, Brazil, between July 2012 and April 2019. Patients not submitted to IVF and/or without AMH level records were excluded. The study included women aged 20-50 years of age submitted to IVF diagnosed with fertility issues.

The patients included in the study were divided into groups based on age and AMH level. The AMH cutoff level was 1.2 ng/mL, as described in the POSEIDON study (Humaidan *et al*., 2016).

The collected data included patient age, start and duration of treatment, diagnosis and reason for infertility, AMH level, number of metaphase II (MII) oocytes retrieved, and IVF outcome. Data points were statistically analyzed based on quantitative, qualitative, partial, absolute, and comparative parameters.

## RESULTS

During the study period, 1,440 patients underwent ART treatment at the clinic. A total of 733 patients aged 20-48 years were enrolled (Table 1) after inclusion and exclusion criteria were considered. However, ART outcomes were available for only 681 of the 733 patients. Live birth rates after 21 weeks of pregnancy were significantly higher among younger patients (aged 35 years and younger). Older patients were more likely to having no oocytes retrieved (*p*=0.0224) (Table 2). All other ART outcomes were similar between the groups.

**Table 1.** Diagnosis of patients seen at assisted human reproduction clinic Conceber during the years 2012 and 2019.

Diagnosis	Patients (n)	Prevalence
Anatomical changes	52	7.09%
Endocrine changes	37	5.05%
Endometriosis	36	4.91%
Male factor	122	16.64%
Ovarian insufficiency	374	51.02%
Idiopathic	112	15.29%
Total	733	100%

**Table 2.** Outcomes per age group of assisted reproductive technology treatments of patients seen at Conceber Clinic in 2012-2019.

Outcomes of ART treatment	Up to 35 years	36 - 39 years	Over 40 years	*p* value
Biochemical pregnancy	48.39% (15)	29.03% (9)	22.58% (7)	0.1869
Complete fertilization failure	31.71% (13)	21.95% (9)	46.34% (19)	0.1567
Live births (>21 weeks)	61.21%A (71)	25.86%B (30)	12.93%C (15)	< 0.0001*
Abnormal/no embryo development	17.65% (9)	41.18% (21)	41.18% (21)	0.0594
Absence of mature oocytes	21.43% (3)	21.43% (3)	57.14% (8)	0.1677
Absence of retrieved oocytes	17.14%A (6)	28.57%AB (10)	54.29%B (19)	0.0224*
Miscarriage (6 to 21 weeks)	37.5% (3)	25% (2)	37.5% (3)	0.8825
Low ovarian response	25% (2)	25% (2)	50% (4)	0.6065
Total cryopreservation	29.53% (57)	36.27% (70)	34.2% (66)	0.5020
Ovarian hyper response	100% (1)	0% (0)	0% (0)	
Ectopic pregnancy	0% (0)	100% (2)	0% (0)	
Other complications	75.00% (3)	25.00% (1)	0%	0.3173
Total	247	232	202	

* Different capital letters between lines in the same column represent significant differences between groups (*p*<0.05).

A total of 717 patients were divided into three age groups (age ≤ 35 years; age between 36 and 39 years; age ≥ 40 years). Mean AMH levels were significantly different between the groups and ranged from 2.65 to 1.35 ng/mL (Table 3).

**Table 3.** AMH levels and number of retrieved and mature oocytes (mean ± standard error) per study group of patients seen at assisted human reproduction clinic Conceber in 2012-2019.

Groups	Patients (n)	AMH level (ng/mL) (Mean ± SE)	Total number of retrieved oocytes (Mean ± SE)	Total number of mature oocytes (Mean ± SE)
Up to 35y	258	2.65±0.16^A^	9.5±0.4^A^	7.14±0.4^A^
36- 39y	251	1.75±0.1^B^	7.17±0.34^B^	5.33±0.34^B^
Over 40y	208	1.35±0.12^C^	5.42±0.36^C^	4.58±0.36^B^

Different capital letters between lines in the same column represent significant differences between groups (*p*<0.05).

The mean number of retrieved oocytes was significantly different between the groups and ranged from 9.5 to 5.42 (Table 3). The mean number of mature oocytes in the patient groups ranged from 7.14 to 4.58 (Table 3). Patients aged ≤ 35 years had a greater number of mature oocytes when compared to individuals in the other groups. There was no significant difference in the number of mature oocytes in the groups of patients aged 36-39 years and of patients aged ≥ 40 years.

The correlations between AMH level, age, and number of retrieved and mature oocytes are shown in Table 4. A negative correlation was observed between age and number of retrieved (r= -0.3354, *p*<0.0001) and mature oocytes (r= -0.2839, *p*<0.0001). AMH level was negatively correlated with age (r= -0.3257, *p*<0.0001). Interestingly, AMH level was positively correlated with number of retrieved (r=0.6702, *p*<0.0001) and mature oocytes (r=0.5770, *p*<0.0001).

**Table 4.** Correlations (r) and significance level (p) between measured response variables.

	Retrieved oocyte	Mature oocyte	Age
**AMH**	0.6702 (< 0.0001)	0.5770 (< 0.0001)	-0.3257 (< 0.0001)
**Age**	-0.3354 (< 0.0001)	-0.2839 (< 0.0001)	1.00

## DISCUSSION

AMH is a glycoprotein secreted by granulosa cells of developing ovarian follicles (Siddiqui *et al*., 2019). It is involved in folliculogenesis and reflects the number of primordial follicles (Seifer & Maclaughlin, 2007; Pellatt *et al*., 2010; Tobler *et al*., 2015). AMH is believed to regulate the number of growing follicles and those that will be selected for ovulation. (Peluso *et al*., 2014). AMH is considered an inhibitor of the early stages of follicular development (La Marca & Volpe, 2006). This means that AMH has an inhibitory effect on initial follicular recruitment, thus preventing the premature depletion of primordial follicles (Durlinger *et al*., 1999; 2001; Kedem *et al*., 2013; 2014; Pankhurst, 2017; Granger & Tal, 2019). AMH has shown to have a strong influence on ovarian function, especially on follicle growth (Rey *et al*., 2000; Visser *et al*., 2006), and has a good correlation with female age, antral follicle count, and ART outcomes (Fanchin *et al*., 2003; Nikolaou & Gilling-Smith, 2004; Nikolaou, 2008; de Vet *et al*., 2019). These findings have allowed the widespread use of AMH in the field of gynecology, from IVF to the diagnosis of different ovarian diseases (Dewailly & Laven, 2019; Bedenk *et al*., 2020).

Ovarian stimulation is an essential step in ART treatments. The administration of exogenous hormones aims at yielding an adequate ovarian response (Broekmans *et al*., 2014; Balachandren *et al*., 2020). Ovarian response measured by the number of retrieved oocytes decreases with age due to progressive ovarian reserve reduction (Hansen *et al*., 2008; Wallace & Kelsey, 2010). Decreases in ovarian reserve are an important cause of female infertility. Low ovarian response may occur in ART in 10-20% of cases and increases with age (Grisendi *et al*., 2019). Therefore, the assessment of ovarian reserve is an extremely important step in IVF cycles with ovarian stimulation, since it allows the identification of patients at risk of low or excessive ovarian response (Alebic *et al*., 2018; Bedenk *et al*., 2020). Once it is not possible to directly determine the number of *in vivo* follicles, indirect measurements of the ovarian reserve can be made through biochemical and ultrasound markers (Broekmans *et al*., 2006). AMH is considered the most sensitive marker for ovarian reserve. AMH offers an accurate, direct measurement of the ovarian follicle pool, and can predict the ovarian response and the number of retrieved oocytes from follicular aspiration (Grisendi *et al*., 2019). AMH concentration assessment offers advantages over other markers such as baseline FSH and antral follicle count, since it is not limited to one phase of the menstrual cycle, does not introduce interobserver variation, and is quite reliable, although there is no internationally standardized trial (Fleming *et al*., 2015; Grossman *et al*., 2017; Tal & Seifer, 2017; Granger & Tal, 2019; Maged *et al*., 2020).

In 2012, the American Society of Reproductive Medicine (ASRM) concluded that AMH was a useful tool for predicting ovarian reserve in patients undergoing IVF treatments, as well as for women at risk for decreased ovarian reserve (Gianaroli *et al*., 2012). The present study showed that AMH levels decrease significantly with age (*p*<0.001). The negative correlation observed in the present study between AMH and age had been reported in the literature. (Nardo *et al*., 2007; Bentzen *et al*., 2013; Keane *et al*., 2017; Loy *et al*., 2017; Massarotti *et al*., 2020). This result was expected, since AMH represents the pool of oocytes existing in the ovary (Nikolaou, 2008; Iliodromiti *et al*., 2014). van Rooij *et al*. (2004) followed 81 patients aged 26 to 45 years for four years. All patients had regular menstrual cycles. Patients who subsequently developed irregular cycles had lower AMH levels than women with regular cycles (0.3 and 1.7 ng/mL, respectively, *p*<0.001). Hansen *et al*. (2008) observed that AMH levels were significantly higher in women of reproductive age when compared to perimenopausal women. Fatima *et al*. (2020) described similar results in a study that looked into the differences in AMH levels in different age groups. The authors reported a significant difference in AMH levels between the studied groups. According to Gomez *et al*. (2016), serum AMH levels decrease annually by approximately 0.384µg/L. In addition, the levels of this hormone are related to the number of oocytes available in the patient. A study by Steiner *et al*. (2011) evaluated several candidate molecules for markers to predict fertility, including FSH, estradiol, AMH, and inhibin B (plasma levels) and FSH and estrone-3-glucuronide (E3G) in the urine of patients. This study included women aged 30-44 years and showed that only AMH was significantly associated with natural fertility. Some authors believe that the decreases seen in AMH levels with aging may be accelerated due to premature ovarian failure and exposure to gonadotoxic chemotherapy (Méduri *et al*., 2007; Dólleman *et al*., 2014; Dunlop & Anderson, 2015).

According to Fatima *et al*. (2020), AMH reflects the ovarian reserve and is a predictor of success for several ART treatments. In patients undergoing ART therapies, serum AMH is the best endocrine indicator of follicular response to ovarian stimulation compared to other commonly used markers, such as FSH, estradiol, inhibin B, and patient age alone (Arce *et al*., 2014; Hawkins Bressler & Steiner, 2018). Compared to FSH, AMH seems to be more strongly associated with age (van Rooij *et al*., 2005; de Vet *et al*., 2019); in addition, AMH levels decrease before increases in FSH can be detected (de Vet *et al*., 2019). Therefore, serum AMH has been considered a more sensitive marker of ovarian reserve than FSH. Although serum AMH levels are controversial for the clinical pregnancy rate in ART, this assessment is still useful to verify the ovarian response to stimulation and to better adjust treatments for patients (Dewailly & Laven, 2019). Age and serum AMH seem to be independent predictors of ovarian reserve and ovarian stimulation outcome in infertile women (Scheffer *et al*., 2018).

According to the ASRM and the European Society for Human Reproduction and Embryology (ESHRE), AMH has the best sensitivity and specificity for measuring ovarian response to controlled ovarian hyperstimulation (Gianaroli *et al*., 2012). A study conducted by Daney de Marcillac *et al*. (2017) found that patients with normal serum AMH levels had better ovarian stimulation, lower cancellation rates, greater numbers of recovered oocytes after follicular puncture, and higher pregnancy rates. The authors also found that patients with low AMH levels had fewer oocytes retrieved. A study by Melado Vidales *et al*. (2017) showed that AMH levels measured during IVF predicted patient ovarian response during the days with follicular growth. The results of the present study support the literature (Fleming *et al*., 2006; Nelson *et al*., 2007; Granger & Tal, 2019; Zhang *et al*., 2019), since patients with higher serum AMH levels showed a significant positive correlation with the total number of retrieved oocytes after follicular puncture. Our data demonstrated that AMH might help predict the number of oocytes produced.

The present study also demonstrated a significant positive correlation between serum AMH and number of mature oocytes. Other authors have suggested that higher serum AMH levels might indicate better oocyte quality, including maturation capacity (Ebner *et al*., 2006; Gomez *et al*., 2016; Borges *et al*., 2017). Although a correlation between embryo quality and chance of blastocyst formation had not been observed, AMH levels were correlated with oocyte quality. In the present study, a significant negative correlation was observed between patient age and number of mature oocytes. AMH levels ranged from 1.35±0.2 to 2.65±0.16 ng/mL. Although AMH levels and number of retrieved oocytes were higher in patients aged 36-39 years than in individuals aged > 40 years, the number of mature oocytes did not differ between these individuals. However, both groups had fewer mature oocytes than patients aged ≤ 35 years. This finding may be related with the median AMH levels seen in the individuals included in this study and explained by the fact that AMH is secreted only by the granulosa cells of preantral follicles and small antral follicles (Maged *et al*., 2020). The mean serum AMH level was approximately 4 ng/mL in young women with good ovarian reserve. However, recently, La Marca *et al*. (2016) described cases with levels below 1 ng/mL and low ovarian response, and levels above 3 ng/mL and high response. According to Ebner *et al*. (2006), oocytes obtained from patients with an intermediate AMH level (between 1.66 and 4.52 ng/mL) have better morphology. For Tal & Seifer (2017), the AMH reference values are age-appropriate and do not refer to a general population of women, regardless of age. These authors considered lower limits of serum AMH of 0.5 ng/mL for women aged 45 years, 1ng/mL for women aged 40 years, 1.5 ng/mL for women aged 35 years, 2.5 ng/mL for women aged 30 years, and 3 ng/mL for women aged 25 years. However, it is important to consider that the ESHRE consensus criteria considered AMH levels < 0.5-1.1 ng/mL as indication of low ovarian reserve (Ferraretti *et al*., 2011). To date, several limits have been suggested for AMH in predicting ovarian response to stimulation. However, there is no uniform agreement due to differences in protocols and populations and AMH test kits (Shahrokhi *et al*., 2018). This means that AMH level results should be used and interpreted with caution.

Patient age is an important factor linked to oocyte quality in IVF cycles. The lower chance of older patients becoming pregnant after IVF is due to poorer response to ovarian stimulation, worse oocyte quality, and higher aneuploidy rates (Franasiak *et al*., 2014; Scheffer *et al*., 2017). Our study showed that the number of retrieved oocytes decreased with patient age (<0.001). Age was negatively correlated with the number of retrieved and mature oocytes. As age advances, an inhibitory effect of AMH on the recruitment of follicles becomes possible (Nelson *et al*., 2009; Pacheco *et al*., 2017).

Regarding the quality of oocytes, some authors believe that a low ovarian reserve might increase the chances of aneuploidy. Several studies suggest that low ovarian reserve and the consequent absence of adequate follicles for selection increase the likelihood of aneuploid oocytes being harvested (Warburton, 1989; Haadsma *et al*., 2010; Rosen *et al*., 2011; Grande *et al*., 2015; Pankhurst, 2017). Total chromosomal non-disjunction and early separation of sister chromatids are correlated with maternal aging. Changes in sister chromatids cohesion may be a causal mechanism that predisposes premature separation and consequently non-disjunction during meiosis. In addition, the asymmetry of the female meiotic division might favor the non-random segregation of chromosomes and chromatids (Johnson *et al*., 2007; Scheffer *et al*., 2018). Oocyte aging causes increased damage to mitochondrial DNA and decreased oxidative phosphorylation and ATP production. Mitochondrial mutations in follicular cells around the oocyte have been correlated with maternal age, suggesting that oxidative phosphorylation in the follicle is compromised (Smeenk *et al*., 2007). Aneuploid oocyte rates increase with age. Women under 35 years of age have an embryo aneuploidy rate of 53% in IVF, while older women present significantly higher aneuploidy rates (74% in patients aged 41-42 years and 93% in women aged 42+ years) (Harton *et al*., 2013).

AMH can be considered an indirect way of measuring the number of preantral and primordial follicles. It is a useful predictor for the pool of follicles that will be ready for final development in the next 3-5 months. Since the number of "selectable" follicles remains approximately constant over time, it can be speculated that AMH levels might predict response to controlled ovarian stimulation. On the other hand, since the number of follicles ready to mature depends on the primordial follicle pool, AMH can be used safely to estimate the ovarian reserve. To date, there is no direct method (except for histological evaluation) for measuring the size of the primordial follicle pool (Gasca *et al*., 2007). However, little is known about the relationship between variations in AMH levels and the qualitative results of ART treatments (implantation, pregnancy, and birth). A recent Brazilian study showed that age negatively affected embryo or blastocyst quality at day 3 (women aged 35+ years) in subjects with low serum AMH levels (<1ng/mL) and antral follicle counts of less than seven (Scheffer *et al*., 2021).

Our data showed that a Brazilian cohort with advanced age had significant decreases in AMH levels and low numbers of retrieved and mature oocytes. However, we found no significant differences in the number of retrieved and mature oocytes between patients aged 36 years or older. We also found a positive correlation between serum AMH levels and the total number of retrieved and mature oocytes. Although this study did not report new data compared to the literature, to our knowledge this is the largest study enrolling Brazilian IVF patients in which correlations between AMH levels, age, and total number of oocytes and mature oocytes from stimulated cycles were described.
